# Evaluation of efficacy and safety for bevacizumab in treating malignant pleural effusions caused by lung cancer through intrapleural injection

**DOI:** 10.18632/oncotarget.22966

**Published:** 2017-12-06

**Authors:** Sun Zongwen, Kong Song, Zhao Cong, Fu Tian, Zhang Yan

**Affiliations:** ^1^ Department of Oncology, Jining No.1 People’s Hospital, Jining, China; ^2^ Department of Respiratory Medicine, Jining No.1 People’s Hospital, Jining, China

**Keywords:** malignant pleural effusion, bevacizumab, platinum chemotherapy, intrapleural injection, meta-analysis

## Abstract

Some clinical investigations have assessed the efficacy and safety of bevacizumab combined with platinum anti-cancer drugs versus platinum drugs alone in treating malignant pleural effusion (MPE) caused by lung cancer through intrapleural injection. This report is a meta-analysis of independent research conclusions. Eleven controlled trials with 769 MPE patients were included in this report. Pooled odds ratios and standardized mean difference with 95% confidence intervals were estimated using the fixed or random effects model of meta-analysis. For treating MPE through intrapleural injection, bevacizumab combined with platinum chemotherapy drugs increased the overall response rate (*p* = 0.003), decreased the incidence of chest pain (*p* < 0.001) and relieved the dyspnea of patients with MPE (*p* = 0.002), as compared with platinum chemotherapy drugs alone. In addition, intrapleural injection of bevacizumab participation decreased the expression of vascular endothelial growth factor in MPE (*p* < 0.001). The main adverse effects of two groups were myelotoxicity, hypertension, digestive reaction and damage of liver and kidney. However, the presence of bevacizumab did not show an extra influence on the incidence of adverse effects (*p* > 0.05). In summary, bevacizumab combined with platinum chemotherapy drugs for treating MPE caused by lung cancer through intrapleural injection has a better benefit of overall response rate and quality of life. And, the participation of bevacizumab did not increase adverse effects.

## INTRODUCTION

Nowadays, research on tumor diseases has made some great progress; malignant pleural effusion (MPE) has become a prominent medical issue due to prolonged survival of cancer patients. Clinically, we often see many patients with advanced cancer accompanied by MPE [1]. In particular, most patients with lung cancer often develop MPE during the course of the disease, which results in a significant decline in the quality of life (QOL) of the patient and leads to a reduction in the expected survival of the patient [2]. Currently, drainage of pleural effusion, thoracic perfusion of chemotherapeutic drugs, and systemic chemotherapy are the primary means of handling MPE [3]. Although MPE is very common, the current understanding of MPE is still limited and there are controversies in almost every aspect of diagnosis and treatment. Recently, the efficacy of pleural perfusion chemotherapy for MPE was reported; it consists of co-administration of cytotoxic drugs (such as cisplatin) and thermotherapy with thoracotomy. However, this method may not be certainly effective for patients with poor pulmonary function and complications [4].

Over-expression of vascular endothelial growth factor (VEGF) has been found in most human tumors, including NSCLC, and is associated with increased tumor recurrence, metastasis, and death [5]. Studies show that two important VEGF tyrosine kinase receptors (VEGFRs), VEGFR1 and VEGFR2, are expressed among vascular endothelial cells of a variety of tumor cells, which bind specifically to VEGF [6]. Binding of VEGF to its cognate receptors leads to phosphorylation of the tyrosine kinase domain, which activates several signaling proteins, including mitogen-activated protein kinase, phosphoinositide 3-kinase (PI3K), and members of the Src family [7]. At present, many molecular signals are found to up-regulate the expression of VEGF such as hypoxia inducible factor 1α (HIF-1α), growth factor epidermal growth factor (EGF), heregulin, tansforming gowth factor-beta 1 (TGF-β), cytokine tumor necrosis factor-α (TNFα) and interleukin-17 (IL-17). Accumulating evidence suggests that VEGF can act directly on cancer cells, affecting different tumor functions, independently of angiogenesis [6].

Bevacizumab is a humanised monoclonal antibody which blocks the binding of circulating VEGF to its receptors. Bevacizumab has been recommended for the first-line treatment of patients with advanced non-squamous non-small cell lung cancer (NSCLC) in combination with carboplatin and paclitaxel [8]. Studies confirm that bevacizumab-based regimens result in a significant effect on survival and response in advanced colorectal, lung, ovarian and kidney cancer [9]. Amazingly, some studies demonstrate that VEGF is associated with the formation of MPE, and VEGF receptor phosphorylation inhibits the formation of MPE in mice with lung adenocarcinomas [10]. Recent years, some clinical studies have specially evaluated the clinical efficacy and safety of bevacizumab in treating MPE. Here, we performed a systematic review and meta-analysis to show whether bevacizumab can be safely and effectively used in the treatment of MPE.

## RESULTS

### Search process of researches

**Figure 1:**
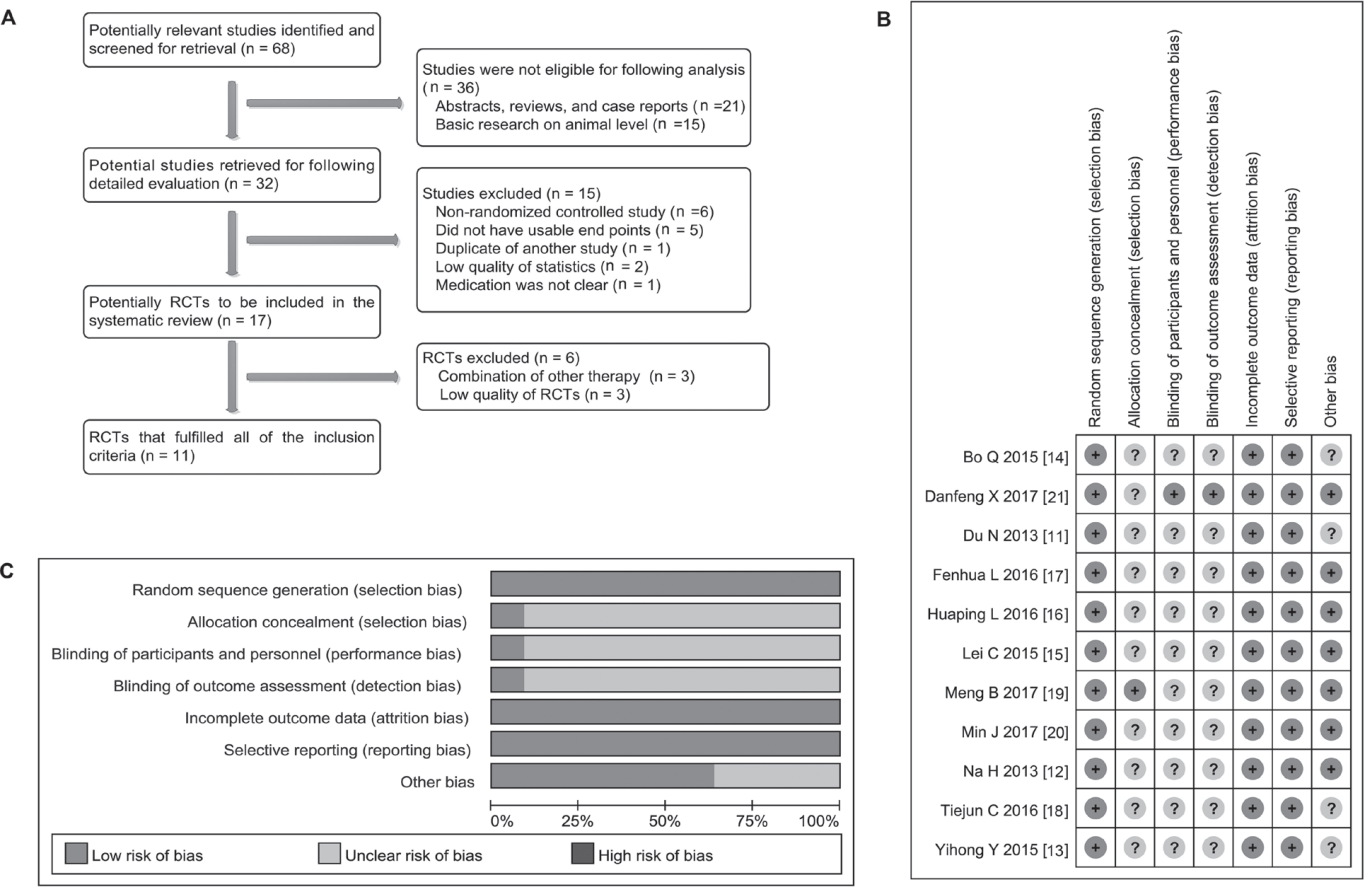
Searching and evaluation of included studies (**A**) Studies were selected from the electronic databases such as PubMed, Embase, Cochrane Library and CNKI database, a total of 11 articles that met the inclusion criteria were included in meta-analysis. (**B**–**C**) Strictly according to criteria made by the Cochrane Handbook, that one study showed the low risk of bias and that 10 investigations displayed the unclear risk of bias.

Initially, 68 studies on bevacizumab with MPE were retrieved. Subsequently, abstracts, reviews and case reports were excluded (21). In addition, 15 studies of animal levels were also abandoned. Of the remaining 32 studies, 15 were removed for the following reasons: non-RCTs (6), no key research indicators were provided (5), repeated published data (1), low quality of statistics (2) and medication was not clear (1). Then, 17 RCTs were considered as suitable for further analysis. However, 6 trials had to be removed because of the following reasons: combination of other therapy (3) and low quality of study (3). Ultimately, 11 studies [11–21] that were fully compliant with the inclusion criteria were included (Figure 1A).

### Clinical features of included studies

As shown in Table 1, a total of 769 patients were involved, including 448 males and 321 females. The age span was from 41 [18] to 82 [11] years old. Of all patients, there were 636 adenocarcinomas (82.7%), 54 squamous cell carcinomas (7.02%), 44 large cell lung cancer (5.72%) and other types of 33 (4.3%). Most of the patients had a moderate to large volume of pleural effusions in these studies. The patient’s QOL was assessed and recorded by karnofsky physical status score (KPS) and eastern cooperative oncology group (ECOG) criterion. The endpoints of these studies were response rate (RR), disease control rate (DCR) and QOL, and most of studies provided the data on adverse effects (AEs) [11–21].

**Table 1:** Data analysis of included studies

Study	*N*	Male	Female	Age (average)	Resource of tumor					Volume of MPE(N)	Quality of Life	Main end point
					LAC	LSCC	SCLC	LCLC	Others			
Du N 2013 [11]	72	44	28	66–82	45	0	0	7	18	−	KPS>60	RR, DCR, QOL, AEs
Na H 2013 [12]	42	27	15	44–68	39	0	0	3	0	−	ECOG>2	RR, DCR, QOL, AEs
Yihong Y 2015 [13]	92	38	54	54.9 ± 10.1	92	0	0	0	0	−	−	RR, DCR, QOL, AEs
Bo Q 2015 [14]	63	36	27	65–78	54	0	0	0	9	Moderate-large	KPS>70	RR, DCR, AEs
Lei C 2015 [15]	54	37	17	43–69	49	0	0	5	0	>1000 ml	-	RR, DCR, QOL, AEs
Huaping L 2016 [16]	84	60	24	−	76	0	0	8	0	−	KPS>60	RR, DCR, QOL, AEs
Fenhua L 2016 [17]	94	50	44	29–65	56	33	0	0	5	−	−	RR, DCR, AEs
Tiejun C 2016 [18]	48	31	17	41–74	48	0	0	0	0	>1000 ml	ECOG>2	RR, DCR, AEs
Meng B 2017 [19]	86	45	41	−	44	21	0	21	0	>1000 ml	−	RR, DCR, QOL
Min J 2017 [20]	52	33	19	57–74	51	0	0	0	1	Moderate-large	ECOG>2	RR, DCR, AEs
Danfeng X 2017 [21]	82	47	35	42–71	82	0	0	0	0	−	−	RR, DCR, AEs

*N*, number of patients; LAC, lung adenocarcinoma; LSCC, lung squamous cell carcinoma; SCLC, small cell lung cancer; LCLC, large cell lung cancer; KPS, karnofsky physical status score; ECOG, Eastern Cooperative Oncology Group; RR, response rate; DCR, disease control rate; QOL, quality of life; AEs, adverse effects.

### Evaluation of clinical and statistical design

As shown in Table 2, in 769 patients, 385 were treated with platinum chemotherapy drugs plus bevacizumab and 384 with platinum chemotherapy drugs alone. The dosage of bevacizumab was administered at 300 mg/time [11, 13, 18] or 5mg/kg/time [12, 14–17, 19–21]. Frequency of administration was 1/week [13, 14, 17, 19–21], 1/2weeks [11, 18] or 1/21 days [12, 15, 16], at least 2 cycles by intrapleural injection after drainage of pleural effusions. There was no difference in clinical features between the two groups (*p* > 0.05), indicating that they had a good comparability [11–21].

**Table 2:** Administration method of included studies

Study	Trial group (*N*)	Control Group (*N*)	Interventions (Groups)		Treatment cycle	Termination of treatment
			Bevacizumab combined with chemotherapy drugs	chemotherapy drugs alone		
Du N 2013 [11]	36	34	Bevacizumab 300 mg + NS 30 mL Cisplatin 30 mg + NS 50 mL	Cisplatin 30 mg +NS 50 mL	1/2 weeks	>3 cycles, or pleural effusion disappeared
Na H 2013 [12]	20	22	Bevacizumab 5 mg/kg + NS 30 mL Cisplatin 75 mg /m^2^ + NS 50 mL	Cisplatin 75 mg/m^2^+NS 50 mL	1/21 days	>1 cycles, or pleural effusion disappeared
Yihong Y 2015 [13]	46	46	Bevacizumab 300 mg/m^2^ + NS 20 mL Cisplatin 75 mg /m^2^ + NS 20 mL	Cisplatin 75 mg/m^2^+NS 20 mL	1/week	>1 cycles, or pleural effusion disappeared
Bo Q 2015 [14]	32	31	Bevacizumab 5 mg/kg + NS 20 mL Cisplatin 40 mg/m^2^ + NS 50 mL	Cisplatin 40 mg/m^2^+NS 50 mL	1/week	>3 cycles, or pleural effusion disappeared
Lei C 2015 [15]	28	26	Bevacizumab 5 mg/kg + NS 30 mL Cisplatin 75 mg/m^2^ + NS 50 mL	Cisplatin 75 mg/m^2^+NS 50 mL	Two times, 1/21 days	>1 cycles, or pleural effusion disappeared
Huaping L 2016 [16]	42	42	Bevacizumab 5 mg/kg + NS 30 mL Cisplatin 75 mg/m^2^ + NS 50 mL	Cisplatin 75 mg/m^2^+NS 50 mL	Cisplatin D1, D3; Bevacizumab D1	1cycle/21D; 4 cycles, or pleural effusion disappeared
Fenhua L 2016 [17]	47	47	Bevacizumab 5 mg/kg + NS 30 mL Cisplatin 45 mg/m^2^ + NS 20 mL	Cisplatin 45 mg/m^2^+NS 20 mL	1/week	>3 cycles, or pleural effusion disappeared
Tiejun C 2016 [18]	24	24	Bevacizumab 300 mg + NS 30 mL Cisplatin 60 mg + NS 50 mL	Cisplatin 60 mg+NS 50 mL	1/2 weeks	>1 cycles, or pleural effusion disappeared
Meng B 2017 [19]	43	43	Bevacizumab 5 mg/kg + NS 30 mL Cisplatin 40 mg + NS 50 mL	Cisplatin 40 mg+NS 50 mL	1/week	>3 cycles, or pleural effusion disappeared
Min J 2017 [20]	26	28	Bevacizumab 5 mg/kg + NS 20 mL Carboplatin 300 mg + NS 50 mL	Carboplatin 300 mg+NS 50 mL	1/week	>3 cycles, or pleural effusion disappeared
Danfeng X 2017 [21]	41	41	Bevacizumab 5 mg/kg + NS 30 mL Cisplatin 60 mg + NS 50 mL	Cisplatin 60 mg+NS 50 mL	1/week	>3 cycles, or pleural effusion disappeared

*N*, numbers of patients; D, day.

### Research quality assessment

As shown in Table 3, all studies were single-center studies [11–21]. Ten studies [11–19, 21] used a randomized approach to group patients. One study [20] did not specify whether a randomized approach was used [20]. Based on the criteria made by the Cochrane Handbook, we evaluated the quality of these studies and found that that one [11] of 11 [11, 13–19, 21] trial (9.09%) showed a low risk of bias and that other studies displayed unclear risk of bias (90.91%) (Figure 1B and 1C).

**Table 3:** Design quality of included trials

Study	Region	Sequence generation	Allocation concealment	Blind	Outcome data	Selective outcome reporting	Other sources of bias	ITT	Risk of bias
Du N 2013 [11]	Single center	Random number table	Unclear	Clear	Yes	No	Unclear	Yes	Low risk of bias
Na H 2013 [12]	Single center	Random number table	Unclear	Unclear	Yes	No	Unclear	Yes	Unclear risk of bias
Yihong Y 2015 [13]	Single center	Random number table	Unclear	Unclear	Yes	No	Unclear	Yes	Unclear risk of bias
Bo Q 2015 [14]	Single center	Random number table	Unclear	Unclear	Yes	No	Unclear	Yes	Unclear risk of bias
Lei C 2015 [15]	Single center	Random number table	Unclear	Unclear	Yes	No	Unclear	Yes	Unclear risk of bias
Huaping L 2016 [16]	Single center	Random number table	Unclear	Unclear	Yes	No	Unclear	Yes	Unclear risk of bias
Fenhua L 2016 [17]	Single center	Random number table	Unclear	Unclear	Yes	No	Unclear	Yes	Unclear risk of bias
Tiejun C 2016 [18]	Single center	Random number table	Unclear	Unclear	Yes	No	Unclear	Yes	Unclear risk of bias
Meng B 2017 [19]	Single center	Random number table	Unclear	Unclear	Yes	No	Unclear	Yes	Unclear risk of bias
Min J 2017 [20]	Single center	Unclear	Unclear	Unclear	Yes	No	Unclear	Yes	Unclear risk of bias
Danfeng X 2017 [21]	Single center	Random number table	Unclear	Unclear	Yes	No	Unclear	Yes	Unclear risk of bias

ITT, intention-to-treat.

### The assessment of heterogeneity

The heterogeneity analysis for all studies showed that chi-squared was 3.17 (Degrees of freedom = 10; *p* = 0.100) and that the statistical value of I-squared (can reflect the degree of heterogeneity) was 0.0%. Statistics test results showed that these studies did not show the heterogeneity. In addition, from the clinical point of view, these studies also had good homogeneity. Based on these results, we used the fixed-effect model to perform the following meta-analysis.

### Intrapleural injection of bevacizumab participation heightened the ORR in treating MPE

**Figure 2:**
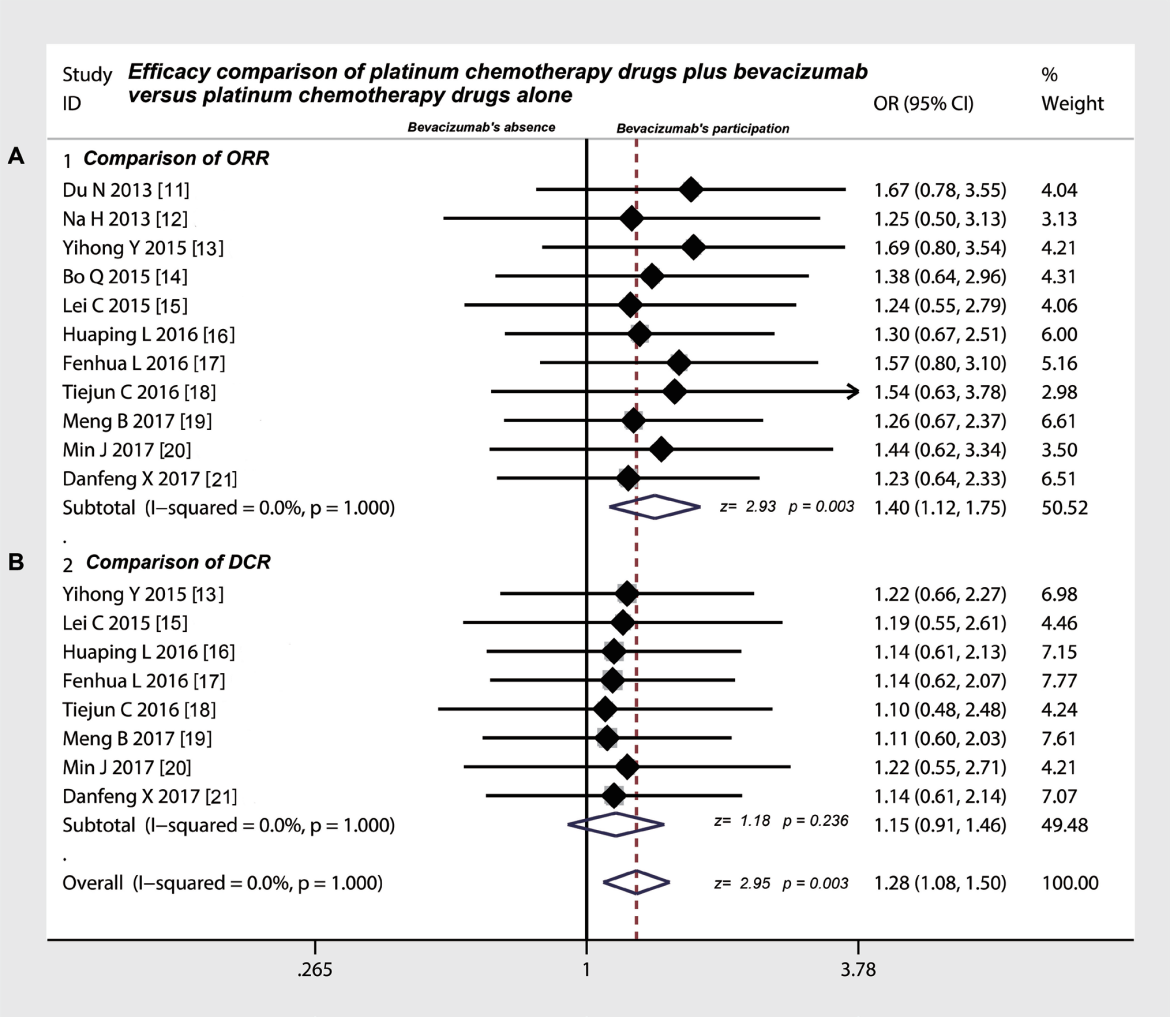
Efficacy comparison between platinum chemotherapy drugs plus bevacizumab and platinum chemotherapy drugs alone by intrapleural injection for controlling MPE (**A**) Intrapleural injection of bevacizumab combination had a higher ORR compared with chemotherapy drugs alone (*p* < 0.05); (**B**) Intrapleural injection of bevacizumab combination had the same DCR compared with chemotherapy drugs alone (*p* > 0.05); ORR, overall response rate; DCR, disease control rate; OR, odds ratio.

As shown in Table 4, eleven studies [11–21] all provided the data on overall response rate (ORR). The fixed effects model of meta-analysis showed that through the way of thoracic perfusion, platinum chemotherapy drugs plus bevacizumab significantly increased the ORR in treating MPE, as compared with platinum chemotherapy drugs alone (odds ratio = 1.40, 95% confidence interval (CI) 1.12–1.75, *Z* value = 2.93, *p* = 0.003) (Figure 2A), which indicated that bevacizumab played a certain effect in treating MPE. In addition, a total of 8 trials [13, 15–21] provided the data on comparing the DCR. However, the statistics test suggested that odds ratio was 1.15 (95% CI 0.91 to 1.46; test for overall effect: *Z* = 1.18, *p* = 0.236) (Figure 2B), which indicated that as to DCR, two different projects did not show significant differences.

**Table 4:** Efficacy of bevacizumab in treating MPE through intrapleural injection

Study	Study design (*N*)		(*N*)		Efficacy of therapy							
			Group 1	Group 2	Group 1				Group 2			
	Group 1	Group 2			CR	PR	SD	PD	CR	PR	SD	PD
Du N 2013 [11]	36	34	Bevacizumab + cisplatin	Cisplatin	17	13	6		2	15	17	
Na H 2013 [12]	20	22	Bevacizumab + cisplatin	Cisplatin	3	14	3		2	13	7	
Yihong Y 2015 [13]	46	46	Bevacizumab + cisplatin	Cisplatin	10	17	12	7	4	6	16	20
Bo Q 2015 [14]	32	31	Bevacizumab + cisplatin	Cisplatin	9	18	5		5	14	12	
Lei C 2015 [15]	28	26	Bevacizumab + cisplatin	Cisplatin	7	17	3	1	4	14	3	5
Huaping L 2016 [16]	42	42	Bevacizumab + cisplatin	Cisplatin	7	28	5	2	4	23	8	7
Fenhua L 2016 [17]	47	47	Bevacizumab + cisplatin	Cisplatin	12	21	9	5	7	14	16	10
Tiejun C 2016 [18]	24	24	Bevacizumab + cisplatin	Cisplatin	11	9	3	1	3	10	9	2
Meng B 2017 [19]	43	43	Bevacizumab + cisplatin	Cisplatin	25	14	3	1	12	19	7	5
Min J 2017 [20]	24	28	Bevacizumab + carboplatin	Carboplatin	6	15	2	1	3	14	5	6
Danfeng X 2017 [21]	41	41	Bevacizumab + cisplatin	Cisplatin	27	11	2	1	19	12	4	6

*N*, cases; Group 1 = bevacizumab combined with chemotherapy drug; Group 2 = chemotherapy drug alone; CR, complete response; PR, partial response; SD, stable disease; PD, progressive disease.

### Intrapleural injection of bevacizumab participation decreased the expression of VEGF in MPE

**Figure 3:**
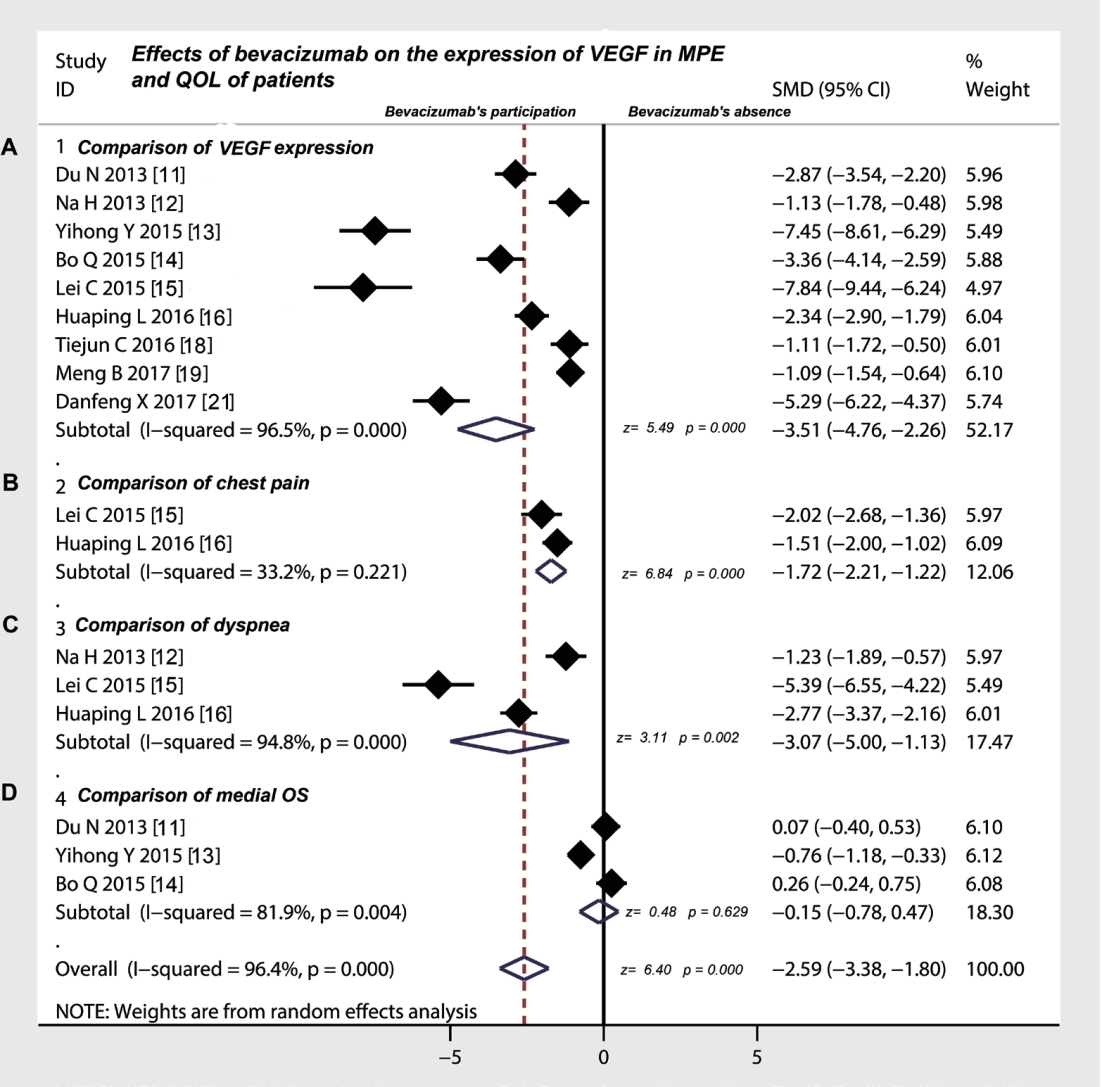
Effects of bevacizumab on the expression of VEGF in MPE and QOL of patients (**A**) Intrapleural injection of bevacizumab combination down-regulated the expression of VEGF in MPE compared with chemotherapy drugs alone (*p* < 0.05); (**B**) Intrapleural injection of bevacizumab combination decreased the incidence of chest pain of patients with MPE compared with chemotherapy drugs alone (*p* < 0.05); (**C**) Intrapleural injection of bevacizumab combination contributed to relieve the dyspnea of patients with MPE compared with chemotherapy drugs alone (*p* < 0.05); (**D**) Intrapleural injection of bevacizumab combination did not shake the median OS of patients with MPE compared with chemotherapy drugs alone (*p* < 0.05); VEGF, vascular endothelial growth factor; QOL, quality of life; OS, overall survival; SMD, standardized mean difference.

As shown in Table 5, 9 trials [11–16, 18, 19, 21] provided the data on VEGF expression in MPE. After intrapleural injection of platinum chemotherapy drugs plus bevacizumab, the expression of VEGF in MPE displayed a down-regulation compared with platinum chemotherapy drugs alone (Figure 3A) (standardized mean difference (SMD) = −3.51, 95% CI was −4.76 to −2.26; test for overall effect: Z = 5.49, *p* = 0.000), which suggested that intrapleural injection of bevacizumab decreased the expression of VEGF in MPE.

### Intrapleural injection of bevacizumab participation reduced the incidence of chest pain in patients with MPE

As shown in Table 5, 2 studies [15, 16] provided the data on the incidence of chest pain of platinum chemotherapy drugs plus bevacizumab versus platinum chemotherapy drugs alone by intrapleural injection for controlling MPE. The statistical test suggested that the odds ratio was –1.72 (95% CI –2.21 to –1.22; test for overall effect: *Z* = 6.84, *p* = 0.0000), indicating that presence of bevacizumab reduced the incidence of chest pain in patients with MPE (Figure 3B).

**Table 5:** Effects of bevacizumab on the expression of VEGF in MPE and QOL of patients with MPE

Study	Study design (*N*)		Expression of VEGF (M ± SD)		EORTC QLQ-C30 evaluation standard (M ± SD)				Follow-up time (Months) (M ± SD)	
	Group 1	Group 2	After treatment		chest pain		Dyspnea		Median OS	
			Group 1	Group 2	Group 1	Group 2	Group 1	Group 2	Group 1	Group 2
Du N 2013 [11]	36	34	15 ± 2	24 ± 4	−	−	−	−	10.3 ± 3.2	10.1 ± 2.9
Na H 2013 [12]	20	22	28 ± 22	60 ± 33	−	−	51.5 ± 4.2	56.3 ± 3.6	−	−
Yihong Y 2015 [13]	46	46	251 ± 31	780 ± 40	−	−	−	−	7.28 ± 2.1	9.25 ± 3.1
Bo Q 2015 [14]	32	31	35.1 ± 12.8	94.6 ± 21.6	−	−	−	−	13 ± 4.2	12 ± 3.6
Lei C 2015 [15]	28	26	18 ± 7	99 ± 13	15.9 ± 4.3	25.2 ± 4.9	7.1 ± 1.8	28.1 ± 5.1	−	−
Huaping L 2016 [16]	42	42	33.6 ± 14.6	71.5 ± 17.6	17.4 ± 6.3	28.7 ± 8.5	9.4 ± 2.9	24.4 ± 7.1	−	−
Tiejun C 2016 [18]	24	24	105 ± 88	194 ± 71	−	−	−	−	−	−
Meng B 2017 [19]	43	43	152 ± 31	259 ± 45	−	−	−	−	−	−
Danfeng X 2017 [21]	41	41	42.6 ± 6.6	88.7 ± 10.4	−	−	−	−	−	−

*N*, cases; Values are given as number of patients (%). VEGF, vascular endothelial growth factor; M ± SD, mean ± standard deviation; EORTC QLQ-C30, European Organization for Research and Treatment of Cancer Quality of Life Questionnaire-C30; Group 1 = bevacizumab combined with chemotherapy drug; Group 2 = chemotherapy drug alone; OS, overall survival time.

### Intrapleural injection of bevacizumab participation contributed to relieve the dyspnea of patients with MPE

As shown in Table 5, three [12, 15, 16] of 11 studies compared the degree of dyspnea on platinum chemotherapy drugs plus bevacizumab versus platinum chemotherapy drugs alone by intrapleural injection for treating MPE. We found that the intrapleural injection of platinum chemotherapy drugs plus bevacizumab contributed to relieve the dyspnea of patients with MPE (Figure 3C), compared with platinum chemotherapy drugs alone (odds ratio = −3.07, 95% CI = −5 to −1.13; *Z* = 3.11, *p* = 0.002). Three [11, 13, 14] of 11 studies compared the median overall survival (OS), however, we did not find any meaningful difference (odds ratio = −0.15, 95% CI was −0.78 to 0.47; test for overall effect: *Z* = 0.48, *p* = 0.629) (Figure 3D)

### Intrapleural injection of bevacizumab participation did not increase the extra AEs

**Figure 4:**
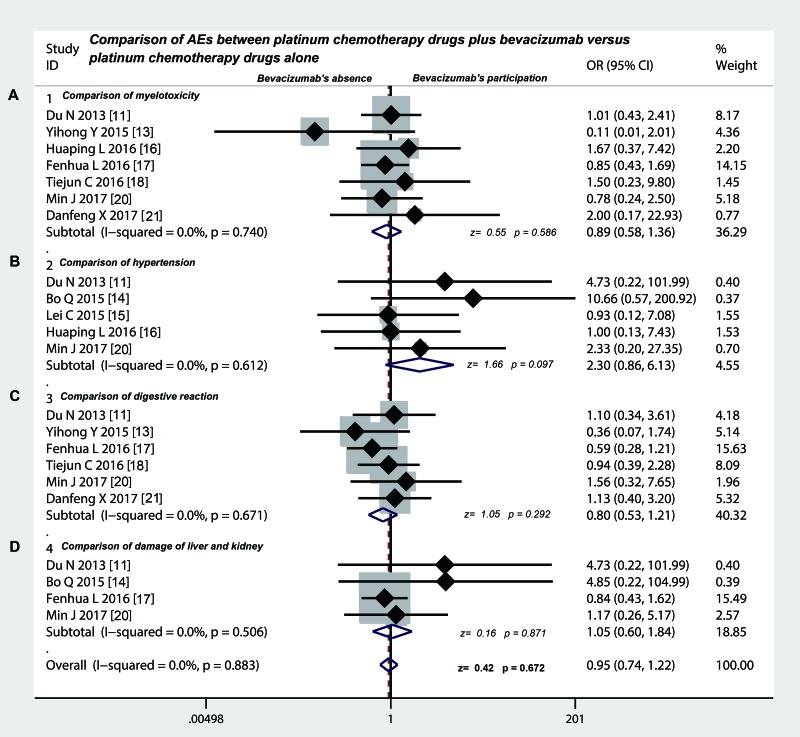
Safety evaluation of platinum chemotherapy drugs plus bevacizumab versus platinum chemotherapy drugs alone by intrapleural injection for controlling MPE (**A**) The bevacizumab combination therapy displayed the same incidence rate of myelotoxicity compared with chemotherapy drugs alone (*p* > 0.05); (**B**) The bevacizumab combination therapy had the same incidence of hypertension compared with chemotherapy drugs alone (*p* > 0.05); (**C**) The incidence rate of digestive reactions in bevacizumab combination therapy group was no difference with chemotherapy drugs alone (*p* > 0.05); (**D**) The incidence rate of liver and kidney damage did not have significant differences between bevacizumab combination therapy versus chemotherapy drugs alone (*p* > 0.05); OR, odds ratio.

As shown in Table 6, the common AEs of platinum chemotherapy drugs plus bevacizumab versus platinum chemotherapy drugs for treating MPE included myelotoxicity (20.7% vs. 16.5%), hypertension (8.24% vs. 3.16%), digestive reaction (31.1% vs. 35.7%) and damage of liver and kidney (20.9% vs. 20.1%). Seven studies [11, 13, 16–18, 20, 21] compared the incidence of myelotoxicity (odds ratio = 0.89, 95% CI 0. 58 to 1.36, *p* = 0.586) (Figure 4A), five studies [11, 14–16, 20] compared the incidence of hypertension (odds ratio = 2.30, 95% CI 0. 86 to 6.13, *p* = 0.097) (Figure 4B), six studies [11, 13, 17, 18, 20, 21] compared the incidence of digestive reaction (odds ratio = 0.80, 95% CI 0. 53 to 1.21, *p* = 0.292) (Figure 4C), four studies [11, 14, 17, 20] compared the incidence of damage of liver and kidney (odds ratio = 1.05, 95% CI 0. 60 to 1.84, *p* = 0.871) (Figure 4D). However, there was no significant difference in the incidence of these AEs between the two groups as mentioned above.

**Table 6:** Comparison of AEs between bevacizumab combined with chemotherapy drug versus chemotherapy drug alone

Study	Myelotoxicity				Hypertension				Digestive reaction				Liver and kidney damage			
	Group 1		Group 2		Group 1		Group 2		Group 1		Group 2		Group 1		Group 2	
	*N*	%	*N*	%	*N*	%	*N*	%	*N*	%	*N*	%	*N*	%	*N*	%
Du N 2013 [11]	15	41.6	14	3.8	2	5.5	0	0	7	19.4	6	16.6	2	5.5	0	0
Yihong Y 2015 [13]	0	0	12	4.3	−	−	−	−	2	26.1	16	34.8	−	−	−	−
Bo Q 2015 [14]	−	−	−	−	5	15.6	0	0	−	−	−	−	2	6.2	0	0
Lei C 2015 [15]	−	−	−	−	2	7.1	2	7.6	−	−	−	−	−	−	−	−
Huaping L 2016 [16]	5	11.9	3	7.1	2	4.7	2	4.7	−	−	−	−	−	−	−	−
Fenhua L 2016 [17]	23	48.9	27	57.4	−	−	−	−	17	36.2	29	61.7	26	55.3	31	66
Tiejun C 2016 [18]	3	12.5	2	8.3	−	−	−	−	16	66.6	17	70.8	−	−	−	−
Min J 2017 [20]	6	25	9	32.1	2	8.3	1	3.5	4	16.6	3	10.7	4	16.6	4	14.3
Danfeng X 2017 [21]	2	4.8	1	2.4	−	−	−	−	9	21.9	8	19.5	−	−	−	−
	*p* > 0.05				*p* > 0.05				*p* > 0.05				*p* > 0.05			

*N*, cases; Values are given as number of patients (%). Group 1 = bevacizumab combined with chemotherapy drug; Group 2 = chemotherapy drug alone.

### Sensitivity analysis and assessment of publication on included studies

**Figure 5:**
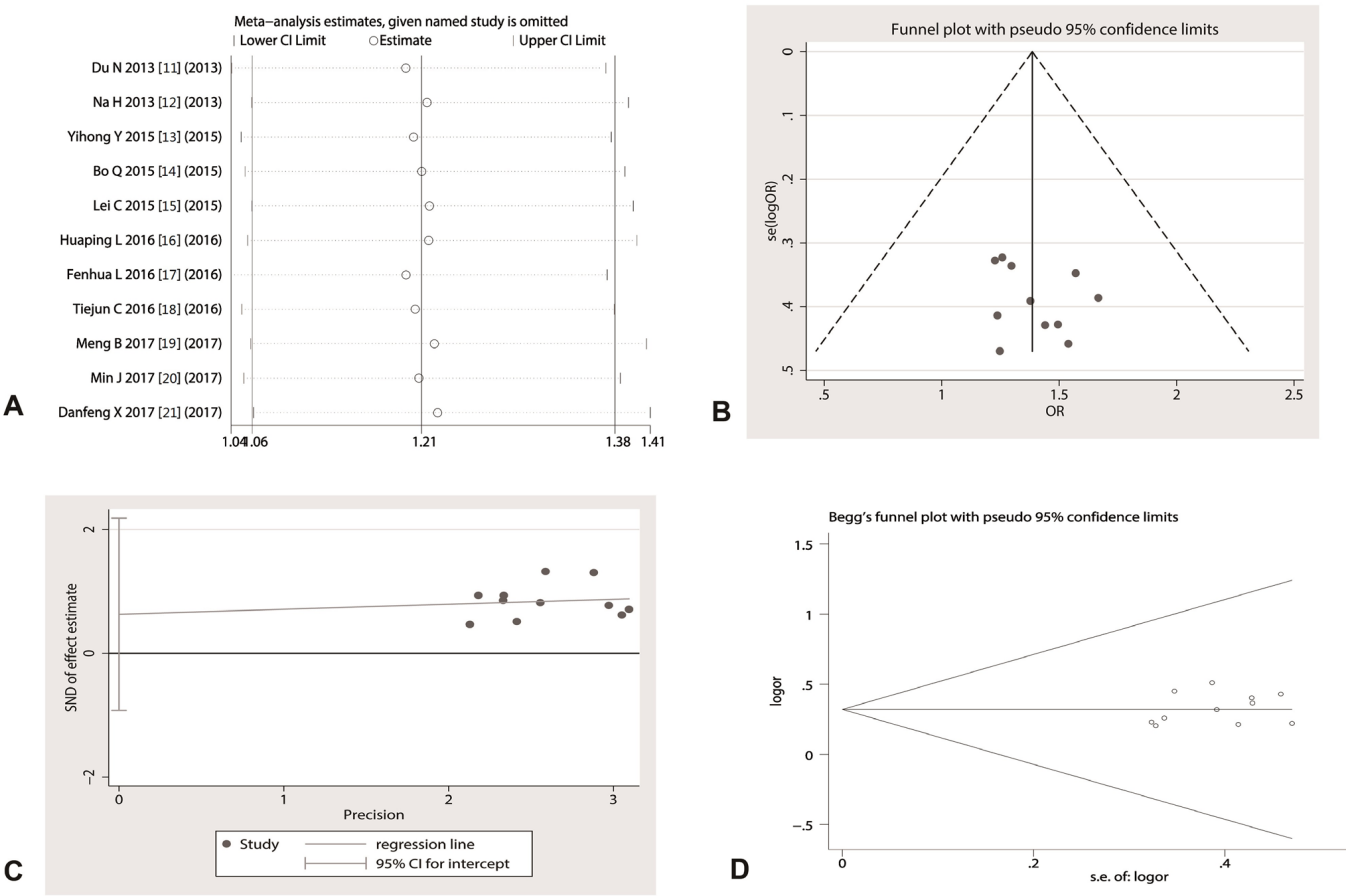
Sensitivity assessment and publication bias analysis (**A**) any of studies did not shake the overall effect of meta-analysis; (**B**) The funnel plot seems to be symmetrical; (**C**) Egger’s test suggested that *p* value was 0.710, indicating the included studies did not exist a publication bias; (**D**) Begg’s test exhibited that *p* value was 0.48, and the shape of funnel plot appeared to be symmetrical.

Sensitivity analysis shows that no single study can change the overall statistical performance alone. The distribution of weights for these studies was 1.23 to 1.69, there was no significant difference in the weight of each study (*p* > 0.05) (Figure 5A). The funnel plot analysis suggested that these studies were evenly distributed on both sides of the funnel plot and were closed to the bottom (Figure 5B). Meanwhile, the statistical results derived from Egger’s test suggested that t value was 0.38 with 16 degree of free (*p* = 0.710) (Figure 5C) and the results of Begg’s test demonstrated that the Std. Dev. of Score was 12.85 (*p* = 0.48) (Figure 5D), which means that the possibility of publication biases in these studies are very small and that the conclusions of the study are more credible.

## DISCUSSION

In clinical practice, approximately 40% of patients with lung cancer will present with or develop malignant pleural effusion (MPE) [22]. Intrapleural injection of anti-tumor chemotherapyagents has been suggested to be used in controlling of MPE because it is believed that when the drug is injected directly into the pleural cavity, its concentration acted on the pleura is much higher than by intravenous injection [2]. Today, there is no cure for metastatic lung cancer, however, some new molecular targeted drugs have been found to be useful in the treatment of lung cancer [3]. In several clinical trials, first-line combination chemotherapies containing bevacizumab are revealed to improve clinical outcomes in patients with advanced non-squamous NSCLC [23]. Bevacizumab is now considered as an essential therapeutic component for eligible patients with stage IV non-squamous NSCLC [24]. These years, some clinical studies reported in China show that platinum chemotherapy drugs plus bevacizumab via intrapleural injection for treating MPE can improve the clinical efficacy and increase the QOL patients with MPE [11–21]. Here, we reviewed 11 studies and performed a meta-analysis to assess whether or not bevacizumab has the potential therapeutic effect to MPE caused by lung cancer via intrapleural injection.

First, we rigorously evaluated the research and design quality of included trials, and found that most of studies had better clinical homogeneity and moderate-higher quality. Second, we conducted a heterogeneous analysis of the included studies because the existence of heterogeneity may lead to instability in meta-analysis conclusions [25]. In our study, we found that these studies did not show the heterogeneity. Therefore, we used the fixed-effect model to perform the following efficacy analysis. We observed that the ORR of platinum chemotherapy drugs plus bevacizumab was significantly higher than that of platinum chemotherapy drugs alone, suggesting that intrapleural injection of bevacizumab combination had a better efficacy compared with platinum chemotherapy alone (odds ratio = 1.40) for controlling the MPE, which reflected an absolute improvement of 22.2%. According to previous study, the bevacizumab has been explored with a 71.4–93.3% MPE control rate through intravenous medication and no significant adverse reactions are cautioned. Bevacizumab has now been approved for first-line treatment in advanced non-small cell lung cancer, and bevacizumab is also recommended for use in cases with MPE [26]. Our conclusion suggested that bevacizumab had a certain effect in treating MPE by intrapleural injection and played a synergistic effect with platinum chemotherapy drugs. We found that the addition of bevacizumab did not increase DCR, but the DCR of two projects was the same, Because the participation of bevacizumab has a high ORR, then the bevacizumab should have an essential application value in controlling MPE.

Over the past several decades, VEGF signaling pathway has been confirmed to be correlated with the angiogenesis of tumors. Bevacizumab is one of the representatives of molecularly targeted drugs targeting this signaling pathway that exerts a unique anti-tumor effect by inhibiting the angiogenesis of tumors [27]. Research shows that bevacizumab developed against VEGF, binds to soluble VEGF, preventing receptor binding and inhibiting endothelial cell proliferation and vessel formation [28]. A series of studies have shown that VEGF levels are reduced during the treatment of bevacizumab and have suggested that VEGF levels can be seen as a marker for the efficacy of bevacizumab [29–31], although the level of evidence is not yet sufficient to utilize it as a standard biomarker. Our findings suggested that intrapleural injection of bevacizumab decreased the expression of VEGF in MPE and thus reflected the therapeutic effect of bevacizumab at the molecular level. We deduce that bevacizumab may directly combined with VEGF in the chest cavity and act on the pleural tissue of high expression of VEGF, thus exert an anti-tumor effect by blocking the expression of VEGF and regulating its function. Today, in terms of the efficacy of cancer treatment, QOL is also considered a key indicator. If the patient gets the same survival time, treatment that can significantly improve QOL is considered to be have more advantages [32]. In our study, we found that intrapleural injection of platinum chemotherapy drugs combined with bevacizumab reduced the incidence of chest pain (1.72 times) and mitigated the dyspnea of patients with MPE (3.07 times), compared with platinum chemotherapy drugs alone, which indicated that intrapleural injection of bevacizumab participation improved the QOL of patients with MPE. Previous study shows that adding bevacizumab to standard chemotherapy in the treatment of advanced non-squamous NSCLC seems to favor a modest improvement in the main outcomes [9, 33] and bevacizumab significantly prolongs OS and progression-free-survival (PFS) when added to first-line platinum-based chemotherapy in patients with NSCLC [34].

The AEs evaluation of drug is very crucial because it relates to the patient’s clinical benefit. Drugs with the same effect, those with small probability of AEs are considered to have a greater advantage. Our analysis showed that regardless of bone marrow toxicity, the occurrence of hypertension, gastrointestinal reactions and liver and kidney damage, the project of platinum chemotherapy drugs combined with bevacizumab had a similar incidence of AEs, as compared with platinum chemotherapy drugs alone. Because of the better efficacy and low incidence of AEs, bevacizumab showed a certain clinical value in the treating patients with MPE. In order to assure the reliability of the conclusion, we did sensitivity analysis and found that any of the studies could not shake the overall statistical effect. In our analysis, the Egger’s tests and the Begg’s tests all implied that the possibility of publication bias in these studies was small. So the conclusions drawn by this meta-analysis should be credible.

Yet, we also found some deficiencies in these studies. Firstly, most of the studies did not perform blindness and allocation concealment, improper performance on these items may exaggerate statistical efficacy. Secondly, the size of most of studies were small, thus affected the statistical efficiency. Thirdly, the vast majority of patients are Chinese (because bevacizumab has been approved to apply on patients with MPE through intrapleural injection), which may lead to geographical and ethnic biases. Despite these shortcomings, our present investigation still suggests a reliable conclusion that MPE patients caused by non-squamous NSCLC can benefit from the intrapleural injection of bevacizumab and will not increase the AEs.

In summary, intrapleural injection of platinum chemotherapy drugs combined with bevacizumab has a better benefit of ORR and QOL in the treatment of MPE caused by NSCLC, which may be involved in the VEGF expression and functional regulation. And, the participation of bevacizumab does not lead to an extra increase of AEs.

## MATERIALS AND METHODS

### Literature search and screening

The RCTs on medical treatment of MPE by intrapleural injection using bevacizumab and platinum chemotherapy drugs were searched from the medical databases including MEDLINE database, EMBASE, Cochrance Library, Chinese Biomedical Literature Database CBM and Chinese Sci-Tech Journals Database (January 2000 to July 2017). The language of the search was limited to English and Chinese. Keywords for searching included: “non-squamous non-small cell lung cancer”, “non-squamous NSCLC”, “malignant pleural effusion”, “MPE”, “cancerous pleural effusion”, “platinum chemotherapy drug”, “thoracic perfusion”, “bevacizumab”, “Avastin”, “randomized controlled trial”, “RCTs”, “cisplatin”, “carboplatin”, and “intrapleural injection”. Two researchers independently searched for literature based on abstracts and keywords and screened the literature. They carefully read each of the selected articles in conjunction with the inclusion and exclusion criteria. When the references that were included in the literature provided the relevant information, we also searched the literature further. When there was disagreement on any study, the third author would intervene to make a decision through negotiation and discussion.

### Inclusion criteria

The following inclusion criteria are strictly enforced: (1) must be clinical control trial for treating MPE caused by NSCLC; (2) must compare platinum chemotherapy drugs plus bevacizumab to platinum chemotherapy drugs alone; (3) cancer cells must be found in pleural effusion; (4) age and gender must not be restricted; (5) the amount of pleural effusion must be moderate to large; (6) must be administered by pleural perfusion; (7) patients were not given systemic chemotherapy or radiotherapy at the same time or within one month; (8) efficacy must be determined by WHO criteria or Response Evaluation Criteria In Solid Tumors (RECIST); (9) adverse reactions (AEs) must be determined by WHO Recommendations for Grading of Acute and Subacute Toxicity; (10) statistical design must be standardized and (11) clinical features between two groups should be better comparable.

### Exclusion criteria

The exclusion criteria are as follows: (1) case reports, abstracts, newsletters, reviews, and conference report were excluded; (2) animal experiments, single arm and observational studies were excluded; (3) patients received other anti-tumor drugs through intravenous and oral administration within one month; (4) thoracic cavity was injected with chemotherapeutic drugs and biological agents within one month; (5) did not provide outcomes of interest; (6) research funding came from drug producers and sellers; (7) ethical account is not clear and (8) descriptions of dosing method and termination time was not clear.

### Collection of key variables

The usual information included: (1) the publication date; (2) patient grouping and counting, gender, ages, histological type, physical status score and pleural effusion volume; (3) administration method, dose and interval; (4) the allocation method and counting of different intervention groups; and (5) the data on study quality. The key outcomes and observation indicators included (1) clinical efficacy and safety, such as complete response (CR), partial response (PR), stable disease (SD), progressive disease (PD), improvement rate of QOL, OS and AEs; (2) the overall response rate (ORR) and disease control rate (DCR); (3) the ORR was defined as CR+PR/overall cases and DCR was calculated as CR+PR+ SD/overall cases; (4) AEs were determined according to the criterion of WHO recommendations and only the incidence of Grade II or above was calculated.

### Efficacy evaluation criterion of the treatment of MPE

The included studies used uniform evaluation criteria of WHO to determine the treatment effect for treating MPE [35]. CR: pleural effusion completely disappeared, and at least 4 weeks or more; PR: pleural effusion was significantly reduced (>50%) and maintained for more than 4 weeks; SD: reduced pleural effusion >50% or increased <25%; PD: pleural effusion increased by >25%. The QOL of the patients was evaluated by the EORTC QLQ-C30 evaluation criteria that contained 30 items, which is a questionnaire developed to assess the quality of life of cancer patients [36].

### Supervision of interventions and methods of administration

Scientific research design: (1) clinical control trial of platinum chemotherapy drugs plus bevacizumab with platinum chemotherapy drugs alone for treating MPE; (2) trial group: intrapleural perfusion of bevacizumab and platinum chemotherapy drugs, the control group: intrapleural perfusion of platinum chemotherapy drugs alone; (3) the dosage of bevacizumab and frequency of intrapleural injection was defined according to the introduction of producer, and at least 2 cycles or pleural effusion disappeared; and (4) outcomes for evaluation: ORR, DCR, QOL, OS and AEs.

### Quality assessment

The research and design quality of included trials was determined according to the criteria provided by Cochrane Handbook (Version 5.0.1), this criteria has been recommended to use for systematic reviews of interventional studies [37]. Based on this criterion, we divided the quality of the included literature into three levels: low risk of bias, risk of bias not clear or high risk of bias. This categorization was defined according to some important items, including random allocation, allocation of hidden cases, whether to perform blindness, whether to describe the withdrawal and loss and whether to perform the intention analysis.

### Statistical analysis

To determine whether there was heterogeneity among the included documents, we used two statistical methods, Chi-square test and *I*^2^ statistic test. If the *P* value of Chi-square test was great than 0.10 and *I*^2^ value was less or equal to 50%, meaning the heterogeneity may not exist, so we selected the fixed effects method. On the contrary, we disclosed the heterogeneity sources and selected the random effects method. For dichotomous variables, we used the odds ratio and the 95% confidence interval (CI) to estimate the statistical effect. For continuous variables, we used standardized mean difference (SMD) and 95% CI. We determined the overall effect using Z-scores, with significance being set at *p* < 0.05. Sensitivity analysis was also performed in which each research was removed from the estimated pool each time in order to determine the impact of this research on overall statistical effect. We employed funnel plot analysis, Egger’s test and Begg’s test to reveal the possibility of publication bias in the included literature. Descriptive statistics of some continuous variables were analyzed by SPSS software (version 20.0; IBM SPSS Statistics, IBM Corporation). Two software, Revman 5.2 (the cochrane collaboration) and Stata version 14.0 (Stata Corporation, College Station, TX, USA) were employed to perform meta-analysis. When the *p* value was less than 0.05, the difference was considered statistically significant.

## Abbreviations

AEs: adverse reactions
CI: confidence intervals
CR: complete response
CNKI: China National Knowledge Infrastructure
DCR: disease control rate
EGF: epidermal growth factor
EORTC QLQ-C30: Europe Organization for Research and Treatment of Cancer, Quality of Life Questionnaire
EMBASE: Excerpt Medica Database
HIF-1α: hypoxia inducible factor 1α
HRQOL: health-related quality of life
IL-17: interleukin-17
ITT: intention-to-treat
KPS: Karnofsky scores
MPE: malignant pleural effusion
NSCLC: non-small cell lung cancer
OR: odds ratio
ORR: overall response rate
OS: overall survival
PD: progressive disease
PFS: progression-free-survival
PI3K: phosphoinositide 3-kinase
PR: partial response
QOL: quality of life
RCTs: randomised controlled trials
RECIST: response evaluation criteria in solid tumors
SD: stable disease
SMD: standardized mean difference
TGF-β: tansforming gowth factor-beta 1
TNFα: tumor necrosis factor-α
US FDA: United States Federal Food & Drug Administration
VEGF: vascular endothelial growth factor
VEGFRs: VEGF tyrosine kinase receptors
